# A homozygous *TARS2* variant is a novel cause of syndromic neonatal diabetes

**DOI:** 10.1111/dme.15471

**Published:** 2024-11-07

**Authors:** Russell Donis, Kashyap A. Patel, Matthew N. Wakeling, Matthew B. Johnson, Masha M. Amoli, Melek Yildiz, Teoman Akçay, Irani Aspi, James Yong, Hanieh Yaghootkar, Michael N. Weedon, Andrew T. Hattersley, Sarah E. Flanagan, Elisa De Franco

**Affiliations:** ^1^ Department of Clinical and Biomedical Science University of Exeter Faculty of Health and Life Sciences Exeter UK; ^2^ Metabolic Disorders Research Centre, Endocrinology and Metabolism Molecular‐Cellular Sciences Institute Tehran University of Medical Sciences Tehran Iran; ^3^ Department of Paediatric Endocrinology, İstanbul University, İstanbul Faculty of Medicine İstanbul Turkey; ^4^ Department of Paediatric Endocrinology Bakırköy Dr. Sadi Konuk Education and Research Hospital İstanbul Turkey; ^5^ Nanavati Super Speciality Hospital Mumbai India; ^6^ Juvenile Diabetes Foundation, Maharashtra Chapter Mumbai India; ^7^ Children and Young People's Diabetes Team St James's University Hospital Leeds UK; ^8^ College of Health and Science University of Lincoln, Joseph Banks Laboratories Lincoln UK

**Keywords:** β‐cells, diabetes, genetic discovery, mitochondrial disease, mitochondrial dysfunction, monogenic diabetes, neonatal diabetes

## Abstract

**Aims:**

Neonatal diabetes is a monogenic condition which can be the presenting feature of complex syndromes. The aim of this study was to identify novel genetic causes of neonatal diabetes with neurological features including developmental delay and epilepsy.

**Methods:**

We performed genome sequencing in 27 individuals with neonatal diabetes plus epilepsy and/or developmental delay of unknown genetic cause. Replication studies were performed in 123 individuals with diabetes diagnosed aged ≤1 year without a known genetic cause using targeted next‐generation sequencing.

**Results:**

Three individuals, all diagnosed with diabetes in the first week of life, shared a rare homozygous missense variant, p.(Arg327Gln), in *TARS2*. Replication studies identified the same homozygous variant in a fourth individual diagnosed with diabetes at 1 year. One proband had epilepsy, one had development delay and two had both.

Biallelic *TARS2* variants cause a mitochondrial encephalopathy (COXPD‐21) characterised by severe hypotonia, epilepsy and developmental delay. Diabetes is not a known feature of COXPD‐21. Current evidence suggests that the p.(Arg327Gln) variant disrupts TARS2's regulation of the mTORC1 pathway which is essential for β‐cells.

**Conclusions:**

Our findings establish the homozygous p.(Arg327Gln) *TARS2* variant as a novel cause of syndromic neonatal diabetes and uncover a role for TARS2 in pancreatic β‐cells.

## Graphical abstract

Using genome sequencing, we identified a homozygous TARS2 variant, p.(Arg327Gln), in four individuals with neonatal diabetes and additional neurological features. This genetic finding highlights an important role for TARS2 in β‐cells.

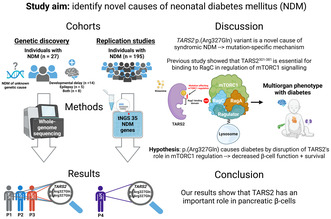

**What's new?**

- What is already known: defining the genetic causes of neonatal diabetes is important for patients' clinical management and can give novel insights into β‐cell biology.
- What this study found: a rare, homozygous *TARS2* variant, p.(Arg327Gln), in four individuals with syndromic neonatal diabetes. We hypothesise that the *TARS2* p.(Arg327Gln) variant results in neonatal diabetes by specifically disrupting TARS2's regulation of the mTORC1 pathway which in turn affects β‐cell survival and function.
- What are the implications of this study: our results establish a *TARS2* variant as a novel cause of syndromic neonatal diabetes which should be tested in individuals with the condition. This genetic finding highlights an important role for TARS2 in β‐cells.

## 1 INTRODUCTION

Neonatal diabetes mellitus (NDM) is a monogenic condition which usually presents before the age of 6 months. There are over 35 known genetic causes of NDM which can either present in isolation or as part of a syndrome. Genetic testing identifies a pathogenic variant in >85% of cases.^1^

Thirteen genetic subtypes of NDM are associated with neurological features, reflecting the critical role of the disrupted genes in the development and/or function of the pancreas and central nervous system (CNS). Specific examples include gain‐of‐function *KCNJ11* and *ABCC8* variants which cause developmental delay, epilepsy, and diabetes through disruption of insulin secretion and neurotransmitter release^2^, ^3^; and loss‐of‐function *PTF1A* variants which cause pancreatic and cerebellar agenesis through defective organ development.^4^

These examples illustrate how identifying genes in which pathogenic variants cause NDM and neurological features highlight biological mechanisms essential for the development and function of both pancreatic β‐cells and neurons.

In this study, we identified a specific *TARS2* variant as a novel cause of NDM, epilepsy and developmental delay highlighting a critical role for TARS2 in the function of β‐cells, and in the CNS.

## 2 RESEARCH DESIGN AND METHODS

We investigated 27 individuals with NDM and neurological features (developmental delay (*n* = 14), epilepsy (*n* = 5) or both (*n* = 8)). In all individuals, pathogenic variants in 35 known NDM genes had been excluded using targeted next‐generation sequencing (tNGS)^5^ (list available on www.diabetesgenes.org).

This study complied with the Declaration of Helsinki with all families providing informed consent. The study was approved by the North Wales Research Ethics Committee (517/WA/0327).

### 2.1 Genome sequencing

Genome sequencing was performed on leukocyte DNA from the 27 probands and 19 sets of parents using the BGISEQ‐500 platform (mean read depth = 34). Reads were aligned to the GRCh37/hg19 human reference genome with BWA‐MEM (v0.7.15) followed by local re‐alignment using GATK IndelRealigner (v3.7.0). Variants were called using GATK haplotype caller and annotated using Alamut Batch (Interactive Biosoftware v1.11, Rouen, France).

We prioritised coding variants and variants affecting the canonical splice sites which matched the following criteria: de novo variants absent in gnomAD_v4,^6^ X‐linked variants absent in males in gnomAD_v4, homozygous and compound heterozygous variants with an allele frequency below 0.001% in gnomAD_v4. All genes with prioritised variants identified in three or more unrelated individuals were followed up.

Variants were assessed in silico using the bioinformatics tools SIFT, PolyPhen‐2 and MutationTaster (accessed through AlamutVisual) and ESM1b.^7^ Prioritised variants were classified using the ACMG guidelines.^8^

### 2.2 Replication studies

The replication cohort consisted of 123 probands with genetically unsolved diabetes diagnosed up to 1 year; 11 individuals had at least one neurological feature (developmental delay (*n* = 7), epilepsy (*n* = 5), microcephaly (*n* = 1)). Samples from all 123 individuals were analysed using a custom‐designed tNGS assay with RNA baits designed for the gene of interest.^5^

### 2.3 Homozygosity and haplotype analysis

Total genomic homozygosity (regions > = 3Mbp) was calculated, and haplotype analysis performed using genome sequencing data or off‐target reads from tNGS data. This allowed us to assess the likelihood of consanguinity within the family and relatedness between people when a variant of interest was identified. This analysis was performed using software developed in‐house^9^ (https://github.com/rdemolgen/SavvySuite).

## 3 RESULTS

**FIGURE 1:**
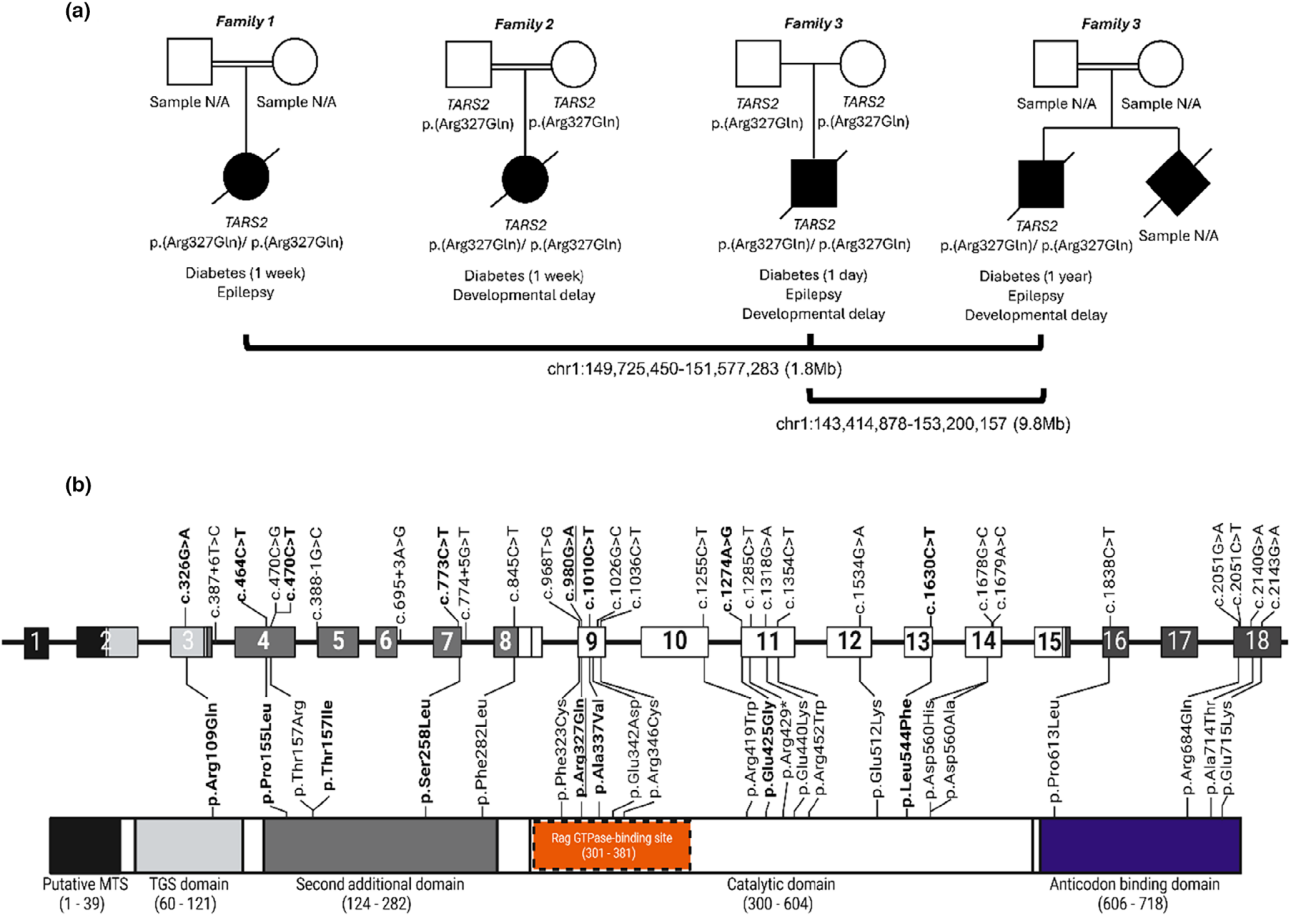
(a) Partial pedigrees and summary of clinical features of the four individuals with the homozygous *TARS2* p.(Arg327Gln) variant. Age at diagnosis of diabetes is given in parentheses. Lines below the pedigree indicate individuals with shared haplotypes. The coordinates and size of the shared haplotypes are shown below the pedigrees. N/A, not available for testing. (b) Reported *TARS2* pathogenic variants. Homozygous variants are highlighted in bold text. The novel homozygous variant identified in this study is underlined. Amino acid changes in the TARS2 protein (bottom) from the corresponding coding variants (top) are joined by vertical slanted lines. Amino acid residue numbers are given in brackets for protein domains. The Rag GTPase‐binding site is highlighted in orange and outlined in dashed lines. All variants are listed according to the NM_025150.4 transcript.

A homozygous missense variant (c.980G>A, p.(Arg327Gln)) in *TARS2* was identified by genome sequencing in three unrelated probands. No further variants that met the filtering criteria were identified. Replication studies identified a fourth individual homozygous for the same p.(Arg327Gln) variant (Figure 1a). No further *TARS2* variants were identified in the replication cohort.

The p.(Arg327Gln) variant is present in gnomAD v4.0.0 in nine heterozygotes (no homozygotes, minor allele frequency 5.58e‐6) and is listed as likely pathogenic in ClinVar (RCV003315462.2). The variant falls within the catalytic domain of the TARS2 protein (Figure 1b) and is predicted to be deleterious by the bioinformatics tools used. The same p.(Arg327Gln) variant had been reported in compound heterozygosity with the p.(Arg419Trp) variant in one individual with Combined oxidative phosphorylation deficiency‐21 (COXPD‐21; OMIM: 615918), a condition characterised by severe hypotonia, epilepsy and developmental delay without diabetes.^10^ Based on this evidence, we classified the p.(Arg327Gln) variant as likely pathogenic, consistent with a diagnosis of COXPD‐21 due to recessively inherited *TARS2* variants in our 4 patients.

Haplotype analysis confirmed that participants 1, 3 and 4 shared a 1.8 Mb region (chr1:149,725,450–151,577,283) encompassing *TARS2*, indicating inheritance of the variant from a distant common ancestor. Participants 3 and 4 shared a larger haplotype of 9.8 Mb (chr1:143,414,878–153,200,157), indicative of closer familial relatedness. Participant 2 did not share a haplotype with any of the other three individuals (Figure 1a).

All four probands were born to unaffected parents, had low birthweight (*Z*‐score range − 4.72 to −1.90) and were diagnosed with insulin‐treated diabetes at a median age of 1 week (range: 1 day–52 weeks). Participant 4 had a younger sibling with hyperglycaemia who died in the neonatal period (sample unavailable for testing). Three probands had a total genomic homozygosity (>1.56%) consistent with consanguinity in the family (Table 1, Figure 1a).

**TABLE 1:** Clinical features of four individuals with the homozygous *TARS2* variant (NM_025150.4:C.980G>A, p.(Arg327Gln)).

		Proband 1	Proband 2	Proband 3	Proband 4
Participant details	Sex	F	F	M	M
	Age at last assessment	4 months±	10 months±	3 months±	19 months±
	Birthweight, gestation (SDS)	N/A, 37 weeks	980 g, 32 weeks (−1.90)	1680 g, 40 weeks (−4.72)	2890 g, 40 weeks (−1.51)
	Cause of death	Seizures and DKA	Not known	Infection	Not known
	Parents related (homozygosity score)	Yes (2.7%)	Yes (3.3%)	No (0.5%)	Yes (6.1%)
	Family History	N/A	N/A	N/A	Sibling with neonatal hyperglycaemia who died. DNA not available for testing.
	Parents' carrier status	Samples not available	Both parents confirmed to be heterozygous	Both parents confirmed to be heterozygous	Samples not available
	Country of referral	Iran	Turkey	India	England
Diabetes	Age at diagnosis	1 week	1 week	1 day	1 year
	Glucose presentation	N/A	N/A	N/A	58.2 mmol/L
	Treatment (dose)	Insulin (not known)	Insulin (0.3 U/kg/day)	Insulin (1.1 U/kg/day)	Insulin (0.3 U/kg/day)
Clinical features	Neurological	Epilepsy	Developmental delay	Developmental delay, epilepsy	Developmental delay, epilepsy
	Muscular	Not reported	Not reported	Not reported	Dystonia, body movement disorder
	Other	Not reported	Umbilical hernia Anaemia Feeding problems Meningitis	Not reported	Renal tubulopathy Sleep disorder Constipation
Imaging/metabolic	Brain MRI	Not reported	Basal ganglion involvement Neurological deterioration Cranial images compatible with mitochondrial disease	Not reported	Not reported
	Metabolic	Not reported	Lactic acidosis	Hypoparathyroidism, metabolic acidosis, hyponatraemia, hypomagnesaemia	Lactic acidosis

Abbreviations: F, female; M, male; MRI, magnetic resonance imaging; N/A, data not available; ±, deceased.

Neurological features were present in all four participants including developmental delay (3/4) and/or epilepsy (3/4). An MRI in participant 2 noted basal ganglion involvement and generalised neurological deterioration, consistent with mitochondrial disease. Lactic acidosis was detected in three participants, one of which also had hypoparathyroidism, hyponatraemia and hypomagnesaemia. Meningitis and anaemia were present in participant 2 with dystonia, body movement disorder and renal tubulopathy reported in participant 4. None of them were treated with mitochondrial supportive therapy. All four children died before 24 months of age (Table 1).

## 4 DISCUSSION

We report the identification of a homozygous *TARS2* variant, p.(Arg327Gln), in four individuals with diabetes diagnosed up to 1 year of age and neurological disease.

Biallelic *TARS2* variants cause COXPD‐21: a clinically heterogeneous condition resulting from a defect in mitochondrial energy metabolism that preferentially affects energy‐demanding tissues.^11^ Within the literature, 28 individuals from 23 families with COXPD‐21 have been described. Developmental delay and axial hypotonia are the most common neurological features reported (in 92% and 85% of individuals, respectively).^10^ Developmental delay was confirmed in 3/4 individuals we report; axial hypotonia was not assessed. Epilepsy was present in 50% of the previously reported cases and was diagnosed in 3/4 (75%) individuals in this study. Diabetes has been described in one individual with COXPD‐21 although the age at diagnosis was not reported.^10^

As a highly metabolically active tissue, the pancreas is dependent on properly functioning mitochondria. Mitochondria provide the ATP required to facilitate insulin secretion from pancreatic β‐cells, and synthesise key metabolites that couple glucose sensing to insulin granule exocytosis.^12^ The most common form of adult‐onset diabetes caused by a mitochondrial defect is maternally inherited diabetes and deafness (MIDD), resulting from a m.3243A>G variant.^13^ More rarely, disease‐causing variants in genes essential for mitochondrial function have been reported to cause neonatal‐onset diabetes. These include biallelic variants in *CYC1* and *NARS2*,^14^, ^15^, ^16^ described in one and two families with syndromic NDM, respectively. Large deletions in the mitochondrial DNA have also been reported in two individuals with Pearson syndrome who presented with NDM.^17^, ^18^ The mechanisms by which these variants lead to β‐cell dysfunction, and consequently diabetes, in these five published families remain unknown.

TARS2 is a class‐II aminoacyl tRNA synthetase. Its canonical role is to ligate threonine to its cognate tRNA molecule during mitochondrial protein translation.^19^ Class‐II aminoacyl tRNA synthetases are essential for mitochondrial protein translation of components of the OXPHOS system and are therefore critical for mitochondrial‐oxidative‐phosphorylation‐mediated ATP production. In vitro studies of *TARS2* variants causing COXPD‐21 showed reduced amino acid activation, tRNA charging activity and dimer formation^20^ as well as decreased levels of OXPHOS system protein components.^21^ The effect of these variants in pancreatic β‐cells has not been established.

TARS2 has a non‐canonical role in the regulation of mTORC1 activation by interaction with Rag GTPases.^20^ The mTORC1 signalling pathway contributes to β‐cell growth, maintenance and survival. A recent study showed that variants in the TARS2^301‐381^ region, including the p.(Arg327Gln) missense variant identified here, decrease binding of TARS2 to Rag GTPases in vitro.^10^ In vivo^22^, ^23^ and in vitro studies^24^ of decreased or abolished mTORC1 signalling in β‐cells found reduced β‐cell mass, defective β‐cell function, hyperglycaemia and impaired glucose‐stimulated insulin secretion. It is therefore possible that, by impairing binding of TARS2 to Rag GTPases, the p.(Arg327Gln) variant decreases mTORC1 signalling, causing reduced β‐cell mass and/or β‐cell dysfunction resulting in NDM. Consistent with this hypothesis, the individual with TARS2‐related disease who had diabetes reported in the literature was also homozygous for a variant in the TARS2^301‐381^ region.^10^ Further studies are needed to assess whether the *TARS2* p.(Arg327Gln) variant causes diabetes through mitochondrial dysfunction, disruption of mTORC1 signalling or both.

In summary, we identified a homozygous p.(Arg327Gln) *TARS2* variant in four individuals with NDM and features of COXPD‐21. These results establish *TARS2* as a novel NDM aetiological gene and highlight a fundamental role for TARS2 in the growth, maintenance and/or survival of human pancreatic β‐cells.

## FUNDING INFORMATION

RD is the recipient of a Diabetes UK PhD studentship (grant 20/0006237). EDF is a Diabetes UK RD Lawrence Fellow (grant 19/0005971) and she is the recipient of an EFSD/Novo Nordisk Future Leaders Award. SEF has a Wellcome Trust Senior Research Fellowship (grant 223187/Z/21/Z). The study was partly funded by a Diabetes UK Project grant (grant 21/0006335). K.A.P. is funded by the Wellcome Trust (219606/Z/19/Z). MJ is a Diabetes UK and Breakthrough T1D (JDRF) RD Lawrence Fellow (23/0006516). ATH is employed as a core member of staff within the National Institute for Health Research–funded Exeter Clinical Research Facility and is an NIHR Emeritus Senior Investigator. This research is supported by the National Institute for Health and Care Research (NIHR) Exeter Biomedical Research Centre (BRC). The views expressed are those of the author(s) and not necessarily those of the NIHR. This research was funded in whole, or in part, by Wellcome (223187/Z/21/Z). For the purpose of open access, the author has applied a CC BY public copyright licence to any Author accepted Manuscript version arising from this submission.

## CONFLICT OF INTEREST STATEMENT

The authors declare no conflicts of interests.
